# Taxonomic information exchange and copyright: the Plazi approach

**DOI:** 10.1186/1756-0500-2-53

**Published:** 2009-03-30

**Authors:** Donat Agosti, Willi Egloff

**Affiliations:** 1 Plazi, Zinggstrasse 16, CH-3007, Bern, Switzerland; 2 Advocomplex, Zinggstrasse 16, CH-3007, Bern, Switzerland

## Abstract

**Background:**

A large part of our knowledge on the world's species is recorded in the corpus of biodiversity literature with well over hundred million pages, and is represented in natural history collections estimated at 2 – 3 billion specimens. But this body of knowledge is almost entirely in paper-print form and is not directly accessible through the Internet. For the digitization of this literature, new territories have to be chartered in the fields of technical, legal and social issues that presently impede its advance. The taxonomic literature seems especially destined for such a transformation.

**Discussion:**

Plazi was founded as an association with the primary goal of transforming both the printed and, more recently, "born-digital" taxonomic literature into semantically enabled, enhanced documents. This includes the creation of a test body of literature, an XML schema modeling its logic content (TaxonX), the development of a mark-up editor (GoldenGATE) allowing also the enhancement of documents with links to external resources via Life Science Identifiers (LSID), a repository for publications and issuance of bibliographic identifiers, a dedicated server to serve the marked up content (the Plazi Search and Retrieval Server, SRS) and semantic tools to mine information. Plazi's workflow is designed to respect copyright protection and achieves extraction by observing exceptions and limitations existent in international copyright law.

**Conclusion:**

The information found in Plazi's databases – taxonomic treatments as well as the metadata of the publications – are in the public domain and can therefore be used for further scientific research without any restriction, whether or not contained in copyrighted publications.

## Background

Global biological diversity is increasingly threatened, but biodiversity conservation and management is in a stiff competition with other vital interests such as food security, economic interest, and space for an increasing human population. More precise knowledge of biodiversity is needed in order to provide convincing arguments for its conservation (CBD [1]; Target 2010 [2]). Despite a simple global scientific naming and classification system for species (the Latin Binomen celebrating its 250th birthday in 2008 in Zoology), despite huge libraries with hundreds of millions of printed pages on natural history, and despite of billions of specimens in natural history collections, access to knowledge of the world's species is cumbersome and inadequate. Finding relevant literature on a given species can be extremely difficult, if not impossible, as neither a comprehensive, global bibliographic database of the publications nor an index to the specific taxonomic treatment of the species exists. For a few groups of species only, such as the ants [3], is there a complete species catalogue and access to digital versions of the related literature available. Without such species catalogues, full text searching of information is impeded, since searches for a particular name tend to result in a huge array of irrelevant data (e.g. mere citations, or other references to topics that are not relevant for the understanding of the description).

The alternative to full text search is to embed domain specific mark-up, such as elements delimiting and identifying scientific names, individual treatments, or materials citations, essentially modeling the logical content. However, marking-up literature after publication can be expensive and time consuming. Costs could be reduced if mark-up could be introduced early in the article production work-flow on manuscripts.

Recently, due to the switch from printed to easily distributed electronic publications, the enforcement of copyright law has become of heightened concern, especially among the publishers in the developed world who produce and sell printed as well as electronic versions of the publications. Subscription act as a barrier to accessing and using this pertinent scientific information, and the development of a truly global knowledge system is impeded. The negative impact of copyright is most obvious in the case of the Biodiversity Heritage Library (BHL [4]), a large scale effort to digitize all the biodiversity literature stored in the large US and UK natural history institutions. BHL does not scan anything that is presumed to fall under copyright: publications that are younger than 65 years and for which no specific copyright waiver has been negotiated will not be included in BHL. Hence much, or even most, information in BHL is, ipso facto, outdated. The more recent publications in biodiversity literature – about 20,000 descriptions of new species each year [5] and an estimated fivefold that number of re-descriptions – are only available to a privileged group of subscribers.

There may be additional limiting factors to the proliferation of worldwide information systems for taxonomic literature. Scientists – and some scientific institutions or organizations – may see their privileged access to such literature as a competitive advantage over those in less privileged, less wealthy circumstances. Often, practicing taxonomists who essentially built up their own libraries during their careers may not see a need for expanded, more open access. Finally, there may still be doubts for some about the feasibility of digitizing the taxonomic literature.

This paper will demonstrate the feasibility of converting taxonomic literature into semantically enhanced XML documents to which multiple human- and machine-readable access is provided. It describes the services and software of Plazi, which provide a fully functional infrastructure that allows adding new publications to be processed by running a semi-automatic mark-up process and storing the enhanced documents into a dedicated server that supports harvesting of its contents. A legal assessment is made of the conversion process as well as an assessment of the status of the various derivatives of the original taxonomic publications created in this process. Because the software components are placed under open-source licenses, the Plazi framework can be operated by organizations like Plazi itself, by publishers, or by other interested parties.

### Plazi.org

Plazi [6] is an independent not for profit organization dedicated to providing access to taxonomic literature by finding ways to remove existing barriers to access and use, such as copyright restrictions. The system is generic and can be adapted for any other domain that describes entities, e.g. for crystallography.

**Figure 1:**
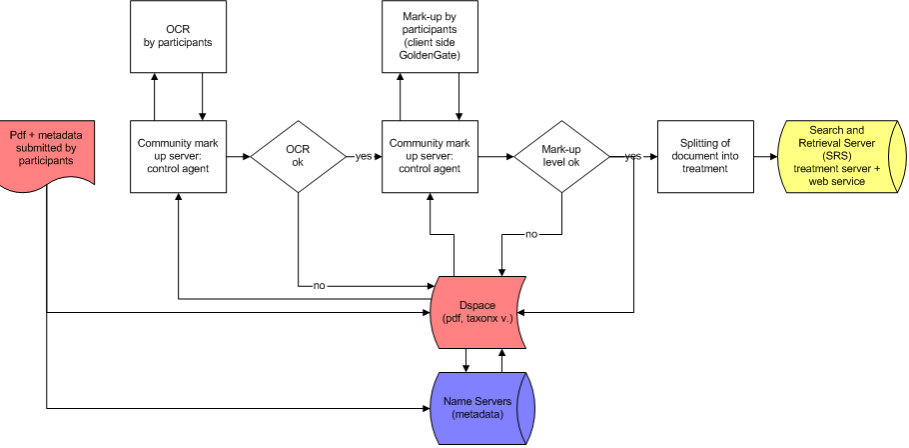
**Plazi workflow**. Red: data that is served; blue: metadata.

The goal of Plazi is to produce semantically enhanced, linked taxonomic documents whose content can be harvested by machines down to the level of granularity the mark-up represents. For that purpose, a workflow has been established that begins with discovering documents that have not yet been included in the system or that are part of a body of publications to be marked-up. If the publication is very recent, the bibliographic metadata is entered into a respective database such as the Hymenoptera Name Server (HNS), from which the data is exchanged with other domain specific databases. HNS then returns the metadata and access points in a standard bibliographic format (Metadata Object Description Schema, MODS) that is included into the XML version of the new publication during the subsequent mark-up process. If the publication is not born digital, the publication is scanned, its text extracted by optical character recognition (OCR-ed) and is saved as image in tiff and pdf formats. The original pdf and its different versions (text, html, XML) are then added to Plazi's DSpace repository (Fig. 1).

At the current stage, mark-up is focused on ants and fish literature in order to build up two bodies of texts that include all the publications of ants from Madagascar from 1758 to 2009 as well as all the approximately 260 fish and ant publications in Zootaxa, resulting in an estimated 12,000 extracted treatments. A detailed description of the development of the Madagascar test body has been accepted for publication [7] and a detailed overview will be published elsewhere. Actually, the repository covers ca. 4'500 publications. In the near future, the Biodiversity Heritage Library will provide digital access to millions of pages of natural history literature, which will be an additional source for our own repository.

TaxonX [8] is an XML schema designed to map the logical structure and content of taxonomic treatments. The schema is, of course, independent of what software may provide valid TaxonX mark-up, but in the present Plazi services, the mark-up process is facilitated by use of our own GoldenGate editor [9]. This provides an incremental process producing ever more fine grained or atomized elements. GoldenGate allows human editors to intervene in the process to correct errors or sub-optimal mark-up. After removing all OCR- and printing artifacts as an initial step, all the taxonomic names are located and marked up using FAT, a specific name recognition algorithm [10]. In succeeding steps, treatments can be subdivided into logical elements. In the best case these can be made to refer to individual observations or specimen data. Similarly the bibliographic references, and the characters and its states in the descriptive elements of the taxonomic treatment can be refined with or without human intervention. These refinements are supported by a "pluggable architecture", allowing Plazi or others to continually improve the automation by the development of software plug-ins written to a published Application Programming Interface (API). During the enhancement process, treatments, materials citation and taxonomic names will be annotated with Life Science Identifier (LSIDs; [11]) that will either be created during the mark-up process or obtained from external services, such as the Hymenoptera Name Server or Zoobank for names, and Bioguid [12] for bibliographic references, or any other service that can be harvested automatically.

Once the mark-up satisfies predefined criteria, the documents are uploaded to the Search and Retrieval Server (SRS). All the marked-up data elements will be saved in respective fields, including the metadata of the publication, and thus guaranteeing the establishment of the provenance of each element. Additionally, the entire TaxonX document is sent to the DSpace repository and administered through an eXist t database [13]. This is a native XML database whose content can be accessed through a human readable interface and by machine. We presently use it for such value-added services as the provision of taxonomic descriptions encoded in the Species Profile Model (SPM [14]).

**Figure 2:**
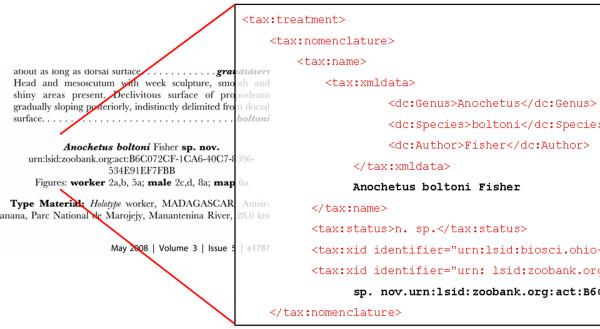
**Sample mark-up page**. Left: sample of an original, published taxonomic treatment. Right: Same treatment marked-up in TaxonX XML schema and enhanced with external identifiers.

Every enhanced XML document can easily be converted into highly customized products such as web pages, pdf documents or prints. The entire document can also be split to parts that can easily be harvested and integrated into websites, e.g. into the Encyclopedia of Life, a major aggregator of species information, which, along with the Global Biodiversity Information Facility (GBIF) supported and participated in the SPM service mentioned above (Fig. 2).

### Data

All the content used in Plazi originates from scientific taxonomic publications, i.e. the publications themselves, particularly their taxonomic treatments and their single materials citations, as well as from external databases like taxonomic name servers, specimen databases, and bibliographic services. For all those elements the source is always cited, including the actual page number and if possible sufficient machine-readable data to allow software to locate the original, or at least a digital copy, of the publication. The act of publishing is one of the key criteria required by the Codes governing biological nomenclature to complete a valid 'nomenclatural act', i.e. to create a valid scientific name for a new discovered species. The publication fulfilling the requirements of a Code is the most authoritative treatment of a particular taxon.

An additional class of data is created to optimize the OCR-process through training the ABBYY Finereader [15] for particular fonts, layouts and particular technological terminology, such as names or morphology. Because many publications exercise rigorous editorial control on these matters, and some became even professional conventions or standards (e.g. species names are always italicized), this class of data is universal in a sense that, once created, it can be used for all related publications, and thus is a prime candidate to be shared among users of the Plazi software framework.

In summary, the information elements managed by the Plazi tools correspond closely to conventions or rules of taxonomic publication. They are, in decreasing order of complexity: the publication, treatments and elements at the same level (e.g. identification keys, synopses); subsection of treatments, such as the nomenclature section, proper description, materials citation, the bibliographic references; and, finally the most basic elements such as the names, the individual materials citations records, a character and its state, or a single bibliographic reference. Metadata for the publication is either created de novo for some new publications, or imported.

### Programs

Except for the commercial ABBYY Finereader all the application programs used by Plazi are open source. This includes both for those supporting our internet services (e.g. DSpace [16], Postgres [17], Simile [18], and eXist [13]) as well as those created by ourselves (GoldenGATE and its plug-ins, SRS), which are licensed under the Berkeley Software Distribution license [19].

### Schemas and transfer protocols

The content of publications needs to be converted into semantically annotated, machine readable documents, which, in the ideal case, are enhanced with references to external resources like taxonomic name servers or bibliographic services. For that purpose we developed the lightweight TaxonX XML schema. Its purpose is to introduce only elements that are unique to taxonomic literature, and not those that could be adopted from other schema.

**Figure 3:**
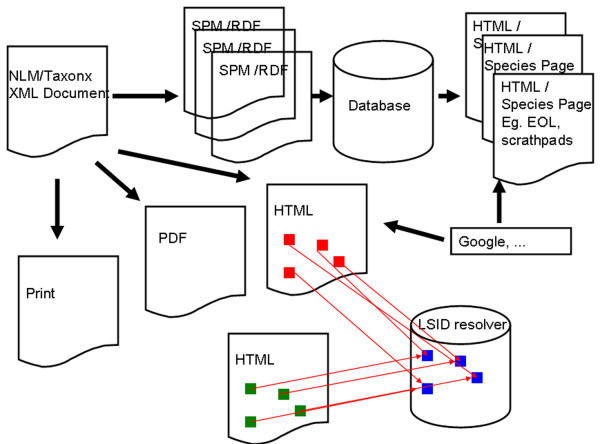
**NLM/TaxonX XML document as source document**. A wealth of derivative products can easily be derived from a NLM/TaxonX XML document, such as print, PDF or HTML products. Furthermore, single treatments can automatically be extracted and used as input for other applications such as Encyclopedia of Life, a typical data aggregator. The presence of Life Science Identifiers (LSID) in the semantically enhanced XML documents allows cross-linking independent web pages through LSID resolvers.

To transfer data to other data providers (Fig. 3), a TAPIR [20] service has been developed to transfer materials citation. More recently, the first SPM application was installed allowing transfer of the content of treatments. An RSS [21] feed allows users to be notified of newly added treatments or materials citations available on the Plazi websites.

For the future a module for the National Library of Medicine publishing schema is being developed [22], allowing the production of taxonomy specific XML documents via an XML based production workflow, and with it the possibilities to convert the documents into many different formats (Fig. 2; see also a NLM/TaxonX marked up publication by Fisher and Smith [23]).

### Legal implications

As described in the previous sections, Plazi workflow aims at transforming printed text into semantically enabled documents from which taxonomic information can be extracted. Species names, treatments and other data as well as bibliographic identifiers are then assembled in a publicly accessible repository.

Questions may occur about the compatibility of this process with existing copyright rules. Is it possible to extract species names and descriptions from protected material without infringing copyright? Is Plazi allowed to make available the assembled data to the interested public?

### Scientific literature and copyright

A great part of scientific literature is protected by copyright; this includes books, articles in scientific journals, sketches, photographs and other forms of publication. If the copyright protection has not yet expired, these works can only be copied, shared and distributed when authorized by individual permission or by broad legal licence.

It is also true that scientific information as such does not qualify as a "literary and artistic work" in the sense of copyright law (art. 1 Berne Convention [24]). The legal term "work" does not mean "text" or "data" or "information". It is differently – and explicitly – defined in national copyright law. The definition of a "literary and artistic work" varies in copyright legislations; it may be described as originality, novelty, individuality, singularity or by other cognate terms. It does not refer to the content, but to the form of presentation (see art. 2 World Copyright Treaty WCT: "Copyright protection extends to expressions and not to ideas, procedures, methods of operation or mathematical concepts as such" [25]). It is the most relevant criteria for qualifying a product as a work: "Work" in the sense of copyright can therefore be defined as an intellectual creation whose form of presentation is original, individual, singular and new.

Databases, in themselves, may constitute such work, but the constituent data within the database, as "facts", are not subject to this definition. "Compilations of data or other material, in any form, which by reason of the selection or arrangement of their contents constitute intellectual creations, are protected us such. This protection does not extend to the data or the material itself..." (art. 5 WCT [25]).

The presentation of names or treatments of species in taxonomic literature is not individual in the sense described above. The content of these treatments may be of high scientific value, it may be singular and new, but it derives fundamental meaning only in the context of scientific conventions that have long been established and practiced. Taxonomic treatments are formulated in a highly standardized language following highly standardized criteria. They adhere to rules and pre-defined logic. They are not "individual", nor "original" in the sense of copyright law. They are thus data, but not "works", and therefore belong to the public domain.

The same applies to biological nomenclature itself. They follow standards established by various Commissions installed by the biological community, including the International Commissions for Zoological Nomenclature ICZN [26], for Botanical Nomenclature (ICBN [27]) and for Fungal Nomenclature (Index fungorum [28]). All these aim to preserve logical schemes and structures that are pre-defined by the scientific community according to pre-established objective criteria. Text written in accordance with such nomenclatural systems is not individual and cannot qualify as work.

The information that users will find in Plazi's Search and Retrieval Server (SRS) – taxonomic treatments as well as the metadata of the publications – is therefore part of the public domain and free of copyright protection. Plazi does not make available protected works from which this material may be extracted. Instead, we present scientific data and by citing the containing material we provide the provenance of each element.

### Extracting scientific works

If there should be a copyright barrier to Plazi, it could only concern the extraction process. Plazi creates its database from taxonomic literature that may be copyright protected. The main copyright question with respect to Plazi is therefore quite simple: Is Plazi permitted to extract data from a protected work?

Traditional scientific efforts consisted in large part exactly of that: Extract information from printed scientific literature, evaluate it, combine it with information extracted from other scientific sources and with new, original research and assemble it into new information. As the distinguished sociologist of science, Robert K. Merton, has noted: "The substantive findings of science are a product of social collaboration and are assigned to the community. They constitute a common heritage in which the equity of the individual producer is severely limited" [29].

This extraction procedure does not involve any copyright barrier: One may read scientific literature, one may excerpt it, one may evaluate it, one may combine it with other information and one may publish the resulting synthesis and new information. All this procedure is just consumption of works, and consumption is none of the prerogatives granted to authors [30,31]. When researchers publish the synthesis of their new ideas, their original research and the gathered information, they cite the sources in which they found the different elements. But this again has nothing to do with copyright, it is an obligation set by scientific rules and academic conventions.

One might assume that the use of machines to follow the same process would merely be an extension of an already accepted practice. However, to read an electronic book or an electronic journal on a screen, one must make a copy; to download a text or a picture from a digital source, one must copy it; to store or print a text, one must create a copy. While reading and excerpting can never be a copyright infringement, as it is only considered consumption of a work, copying poses a different problem. It is one of the many uses of works that requires an authorization, whether an individual permission or a legal licence (art. 9 Berne Convention [24]).

### Workflow based on legal licences

As the Plazi workflow includes the reproduction of documents, is there thus a requirement for such an authorization? Works are scanned, they are semi-automatically marked-up and they are processed by algorithms in order to make extraction of names, treatments and finer grained information possible. Texts or pictures will repeatedly be reproduced during this process. For Plazi to be fully effective, it must be able to operate against the full body of taxonomic literature. At our projected scale of operation, it is not technically practicable to seek individual permissions on a case-by-case basis. The process concerns millions of documents. Neither can the extraction process be limited, say, to documents published under a copyright waiver. The only feasible solution is to work on the basis of legal licences.

The legal licence upon which Plazi workflow is chiefly based has been introduced in Swiss copyright law in 2008 (art. 24a Swiss Author's Rights Law [32]). It allows temporary acts of reproduction, when the copies are transient or incidental, and are an integral and essential part of a technological process, as far as the purpose is to enable a lawful use of the works. In addition, the act of reproduction, allowed by this legal licence may not have an independent economic significance. These criteria correspond to art. 9(2) Berne Convention [24] and art. 10 WCT [25]. There are similar copyright exceptions in most European countries, implementing art. 5 (1) of the European Directive 2001/29/EC of 22 May 2001 on the harmonisation of certain aspects of copyright and related rights in the information society [33]. On a different legal basis, the "fair-use-practice" in US copyright law, leads to comparable results.

The second legal licence concerns the use of works for internal information and documentation (art. 19 Swiss Author's Rights Law [32]). It allows one to download and to reproduce protected works for internal use in administrations, public and private bodies and other institutions.

The Plazi workflow is conceived following these Swiss copyright rules: Works are copied several times during the mark-up and the extraction process, but the copies are only transient. As a result of this process, Plazi presents scientific data and metadata from original sources but not the works themselves. Literary and artistic works such as scientific publications, photographs or illustrations are not made available to the public. They remain restricted to internal use as long as they are stored only for the mark-up and extraction process. No further use is made of the transient copies that have been used for the extraction process. Therefore, the Plazi workflow is covered by the mentioned legal licences.

However, we consider this situation as provisional and inadequate. Plazi advocates enlarging copyright exceptions or legal licences in order to make scientific knowledge further available. There is a huge public interest in getting free access not only to text and data, but also to scientific illustrations [34,35]. Such extensions will make scientific knowledge available to all. The public interest justifies serious consideration of further copyright exceptions for scientific purposes [36].

### Organisation under Swiss copyright law

That Plazi rules refer to Swiss copyright law is primarily justified by the fact, that Plazi is based in Switzerland. However, this choice is not merely incidental. Switzerland is also the host country of the basic international copyright treaty ("Berne Convention") as well as the seat of the World Intellectual Property Organisation WIPO.

The most relevant aspect of this choice is the fact that there is no legal database protection in Switzerland as it exists in EU-countries. This legal instrument, laid down in the Directive 96/9/EC of 11 March 1996 on the legal protection of databases [37], protects databases, "which show that there has been qualitatively and/or quantitatively a substantial investment in either the obtaining, verification or presentation of the contents" through a so called "sui-generis-right". This right allows preventing extraction and/or re-utilization of the whole or of a substantial part of the contents of that database.

One should not confound this specific database protection with copyright protection. Copyright only protects databases, "which by reason of the selection or arrangement of their contents constitute intellectual creations (...). This protection does not extend to the data or the material itself" (art. 5 WCT [25]). The very essential difference is, that the sui-generis-right does not protect the form of presentation. It prevents extraction and/or re-utilization of content. This is in contradiction to the basic principles of copyright, which protects the form of presentation, not content.

This European Database Directive is therefore a serious obstacle to scientific information exchange. That's why Plazi organizes its work in a way that excludes the application of European database protection. The whole workflow, as well as the storage of documents, is based on Swiss law, which does not provide such particular database protection.

## Conclusion

In conclusion, Plazi workflow is absolutely compatible with copyright protection. During the extraction process, only transitory copies are produced which serve exclusively to a lawful use and are destroyed or restricted to internal use, as soon as the extraction process is completed. This practice is fully compliant with law. The results of the extraction process, i.e. taxonomic information such as species names and treatments as well as metadata and bibliographic references, are not protected by copyright because the information is not work. This material belongs therefore to the public domain and can be used without any restriction.

The massive legacy of taxonomic publications offers a huge amount of data that is relatively well structured in the taxonomic domain. However, creating access incurs a huge cost that at the moment is insurmountable. Though we have designed tools that can speed up this transition, and though, as we argue here, there should be no legal obstacles prohibiting open access, the future lies clearly in prospective changes to our current publishing model. Publishing semantically enhanced descriptions using a domain specific publishing XML schema will open up this important body of literature and make it a logical starting point for identifying and accessing data on particular organisms, since links to all the underlying data and citations are provided. From the point of view of the international Conservation Commons [38], it seems to be extremely important that all the barriers to access and use be removed, so that harvesting and exchange of scientific data is possible immediately upon publication.

## Competing interests

The authors declare that they have no competing interests.

## Authors' contributions

DA a professional taxonomist specializing in ants, participated from the very beginning in the development of Plazi. He drafted the introduction and the description of Plazi. WE a professional lawyer specializing in copyright, joined the Plazi group as a legal consultant after its formation. He drafted the section on legal implications. The authors jointly reviewed and approved the final manuscript.
